# Intramural Outflow Tract Ventricular Arrhythmia With Preferential Conduction Mimicking a Left Ventricular Summit Origin

**DOI:** 10.1016/j.jaccas.2026.107269

**Published:** 2026-03-14

**Authors:** Junji Maeda, Yousaku Okubo, Hiroshi Oe, Shunsuke Ishida, Naoki Ishibashi, Shogo Miyamoto, Sho Okamura, Takehito Tokuyama, Noboru Oda, Yukiko Nakano

**Affiliations:** Department of Cardiovascular Medicine, Hiroshima University Graduate School of Biomedical and Health Sciences, Hiroshima, Japan

**Keywords:** coronary venous mapping, double balloon technique, ethanol ablation, intramural myocardium, preferential conduction, ventricular arrhythmias

## Abstract

**Background:**

Intramural ventricular arrhythmias with preferential conduction are challenging to ablate because the breakout site may be remote from the true site of origin.

**Case Summary:**

A 61-year-old man presented with drug-refractory premature ventricular contractions after failed catheter ablation. Electrocardiography suggested an epicardial outflow tract origin. Endocardial mapping identified a prepotential at the left-right coronary cusp junction with a perfect pace map but an extremely long stimulus-to-QRS latency, indicating activation via a preferential conduction pathway. Coronary venous mapping with a microelectrode catheter revealed an earlier prepotential within a septal perforator vein, consistent with an intramural source. Because endocardial ablation was ineffective, selective retrograde venous ethanol ablation using a double-balloon technique was performed, resulting in immediate and durable elimination of premature ventricular contractions without complications.

**Discussion:**

Recognition of intramural substrates with preferential conduction is essential for selecting effective ablation strategies.

**Take-Home Messages:**

Intramural ventricular arrhythmia with preferential conduction can be difficult to ablate because the breakout site from the conduction pathway may be remote from the true site of origin, leading to misleading electrocardiographic and endocardial mapping findings. Coronary venous mapping can identify intramural substrates and guide selective therapies, such as double-balloon–assisted ethanol ablation, when conventional endocardial ablation fails.

## Visual Summary

**Figure:**
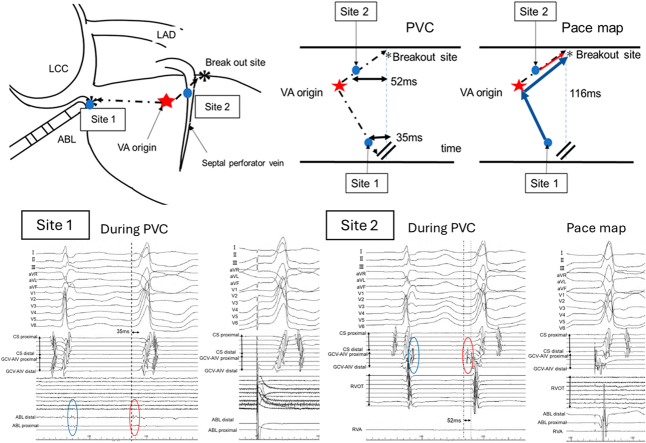

## History of Presentation

A 61-year-old man was referred to our institution for management of symptomatic, drug-refractory ventricular arrhythmia (VA) after a failed catheter ablation at another hospital. He reported frequent palpitations and exercise intolerance. Physical examination was unremarkable, with stable vital signs and no signs of heart failure.

**Take-Home Messages**

• Intramural ventricular arrhythmia with preferential conduction can be difficult to ablate because the breakout site from the conduction pathway may be remote from the true site of origin, leading to misleading electrocardiographic and endocardial mapping findings.
• Coronary venous mapping can identify intramural substrates and guide selective therapies, such as double-balloon–assisted ethanol ablation, when conventional endocardial ablation fails.

## Past Medical History

The patient had no history of structural heart disease, cardiomyopathy, or coronary artery disease. There was no family history of sudden cardiac death.

## Differential Diagnosis

The differential diagnosis included idiopathic outflow tract VA, epicardial ventricular arrhythmia arising from the left ventricular outflow region, and intramural VA with preferential conduction.

## Investigations

**Figure 1:**
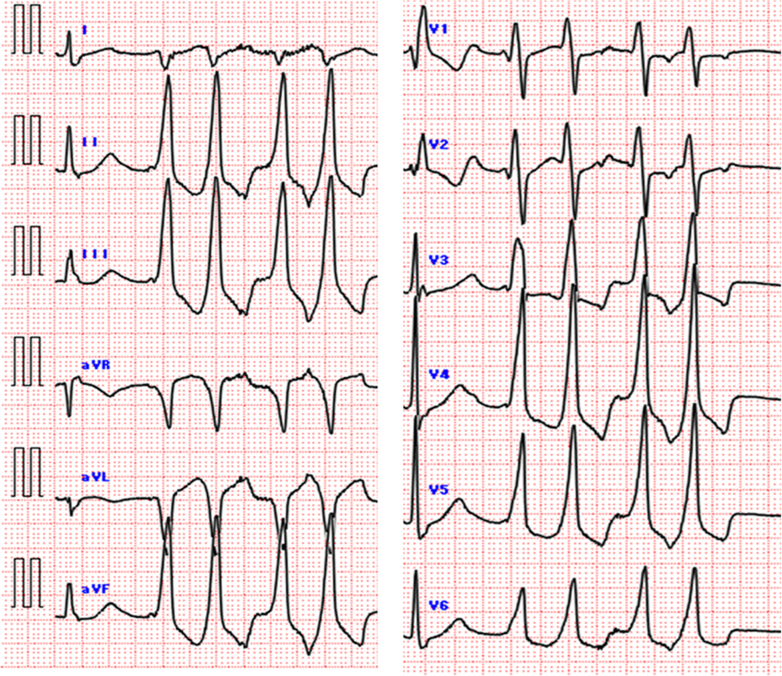
12-Lead Electrocardiogram of the Clinical Premature Ventricular Contractions The 12-lead electrocardiogram shows premature ventricular contractions with a QS pattern in lead I, an inferior axis, and a nonspecific intraventricular conduction delay–like morphology. Precordial transition occurs early at lead V_2_. The R-wave amplitude ratio of lead III to lead II is 1.29, and the Q-wave amplitude ratio of aVL to aVR is 1.6. The maximum deflection index is 0.54. These electrocardiographic features are suggestive of an outflow tract ventricular arrhythmia with an apparent epicardial pattern.

Twelve-lead electrocardiogram during premature ventricular contractions demonstrated a QS pattern in lead I with an inferior axis and an early precordial transition at lead V_2_ (Figure 1). Twenty-four-hour Holter monitoring revealed frequent premature ventricular contractions (PVCs) with a burden of 32.9% and runs of nonsustained ventricular tachycardia up to 12 beats. Transthoracic echocardiography showed normal cardiac structure and preserved left ventricular function. Cardiac magnetic resonance imaging demonstrated normal biventricular size and systolic function, with no regional wall motion abnormalities and no late gadolinium enhancement. Positron emission tomography/computed tomography showed no abnormal myocardial uptake, excluding active myocardial inflammation. Twelve-lead electrocardiogram suggested an epicardial outflow tract origin.

## Management (Medical/Interventions)

**Figure 2:**
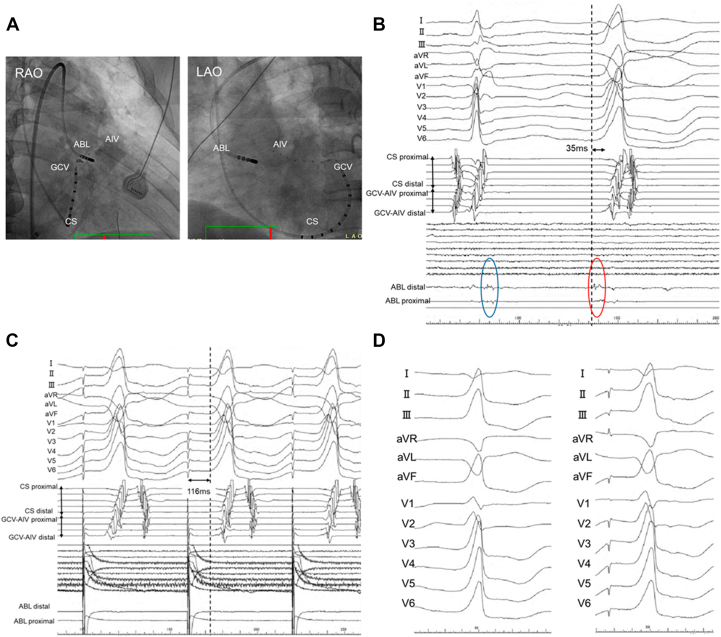
Endocardial Mapping at the Left and Right Coronary Cusp Junction (A) Fluoroscopic images during endocardial mapping show the ablation catheter positioned at the junction of the left and right coronary cusps (RAO and LAO views). (B) Intracardiac electrograms during premature ventricular contractions demonstrate a discrete prepotential preceding QRS onset by 35 ms (red oval); during sinus rhythm, a delayed far-field potential is observed at the same site (blue oval). (C) Pace mapping from this site shows an extremely prolonged stimulus-to-QRS latency of 116 ms. (D) 12-lead electrocardiograms during pace mapping closely match the clinical premature ventricular contractions. ABL = ablation catheter; AIV = anterior interventricular vein; CS = coronary sinus; GCV = great cardiac vein; LAO = left anterior oblique; RAO = right anterior oblique.

Antiarrhythmic drugs, including amiodarone, mexiletine, and bisoprolol, were discontinued for more than 2 weeks before the procedure. The earliest endocardial activation was identified at the junction of the left and right coronary cusps (LCC/RCC), where a discrete prepotential preceded the QRS onset (Figures 2A and 2B). Pace mapping from this endocardial site produced a generally good match with a markedly prolonged stimulus-to-QRS latency, suggesting a remote intramural source (Figure 2C). However, subtle differences were observed compared with the clinical PVC, including slightly higher R-wave amplitudes in leads V_1_ and V_2_ (Figure 2D). Radiofrequency ablation was performed using an irrigated contact force–sensing catheter with a power setting of 35 W for 180 seconds from both the right ventricular outflow tract and the LCC/RCC junction; however, the PVCs were not suppressed.

**Figure 3:**
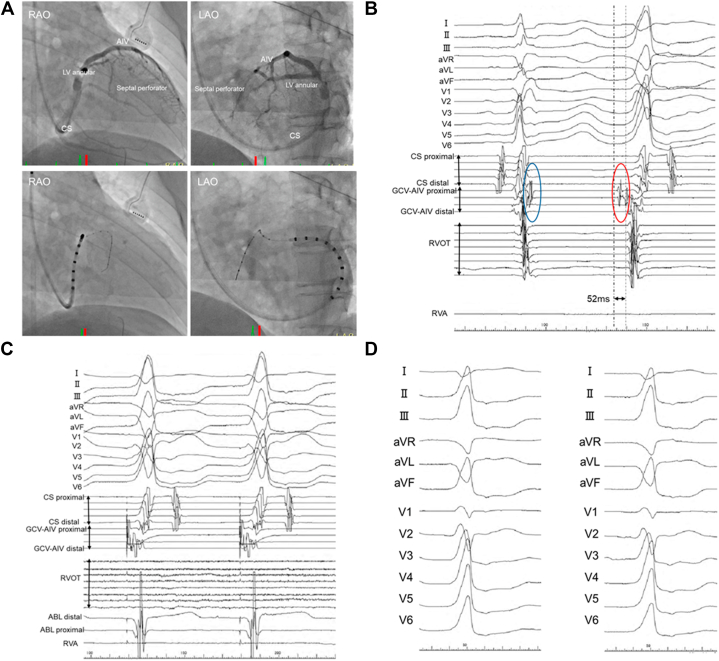
Coronary Venous Mapping Identifying the Intramural Arrhythmogenic Substrate (A) Coronary sinus venography and branch mapping in RAO and LAO views demonstrate catheter positioning within the coronary venous system. (B) Electrograms recorded from a septal perforator vein show the earliest activation, with a sharp prepotential preceding QRS onset by 52 ms during premature ventricular contractions (red oval) and delayed near-field potentials during sinus rhythm (blue oval). (C) Pace mapping from this site reproduces the clinical morphology with a stimulus-to-QRS latency of 52 ms. (D) 12-lead electrocardiograms during pace mapping closely match the clinical premature ventricular contractions. LV = left ventricle; RVA = right ventricular apical; RVOT = right ventricular outflow tract; other abbreviations as in Figure 2.

**Figure 4:**
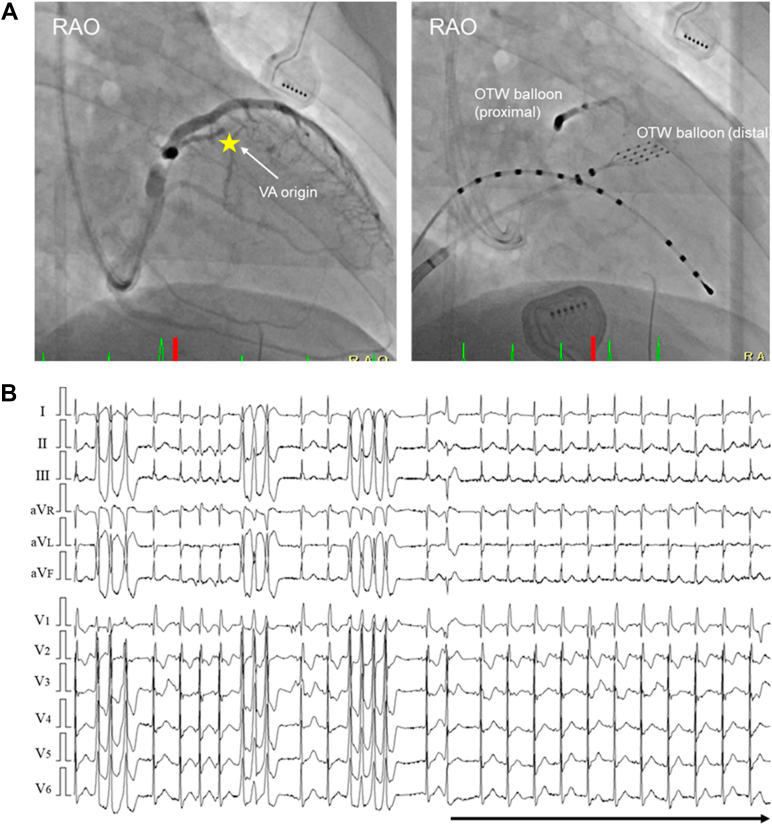
Double Balloon–Assisted Retrograde Venous Ethanol Ablation (A) Coronary sinus venography and fluoroscopic images show double-balloon occlusion of a septal perforator vein using over-the-wire balloons, with a 1.5-mm distal balloon and a 3.0-mm proximal balloon, allowing selective ethanol delivery while preventing distal spread toward the atrioventricular conduction system. (B) 12-lead electrocardiogram demonstrates immediate elimination of premature ventricular contractions after injection of a total of 3.0 mL of 98% ethanol, confirming successful ablation of the intramural substrate. OTW = over the wire; RAO = right anterior oblique; VA = ventricular arrhythmia.

Given these findings, the coronary venous anatomy was reassessed. An over-the-wire microelectrode catheter advanced into a septal perforator vein branching from the left ventricular annular vein recorded an earlier prepotential, and pace mapping reproduced the clinical morphology with a similar latency, indicating direct capture near the intramural focus (Figure 3). Selective venography of the septal perforator vein was performed for anatomical confirmation; however, contrast injection alone did not suppress the PVCs. Because effective lesion delivery from the endocardial surface was considered unlikely, selective chemical ablation was performed. Using 2 internal jugular venous sheaths, a 1.5-mm over-the-wire balloon was positioned distal to the target site, and a 3.0-mm over-the-wire balloon was positioned proximally to achieve complete venous occlusion, followed by controlled injection of 98% ethanol (total volume: 3.0 mL) (Figure 4A). The PVCs were immediately suppressed after minimal ethanol delivery, resulting in complete elimination (Figure 4B).

## Outcome and Follow-Up

The PVCs were completely eliminated immediately after ethanol ablation, with no recurrence during the procedure or under pharmacological provocation. No procedural complications, including atrioventricular conduction disturbance or coronary venous injury, were observed. The patient's symptoms resolved promptly after ablation. During the 1 year of follow-up, ambulatory electrocardiographic monitoring demonstrated sustained suppression of VA, with no recurrence of PVCs or nonsustained VA, and the patient remained asymptomatic without antiarrhythmic drug therapy.

## Discussion

This case highlights the diagnostic and therapeutic challenges of VA arising from an intramural substrate with preferential conduction.^1^^,^^2^ In such cases, the myocardial breakout site may be spatially remote from the true site of origin, leading to misleading electrocardiographic features and failure of conventional endocardial ablation.

In our patient, surface electrocardiography suggested an epicardial outflow tract origin, a pattern commonly attributed to left ventricular summit arrhythmias.^3^^,^^4^ However, detailed electrophysiological assessment revealed key findings inconsistent with a purely epicardial source. At the LCC/RCC junction, a discrete prepotential preceded QRS onset, and pace mapping produced a generally good match with an extremely prolonged stimulus-to-QRS latency.

**Figure 5:**
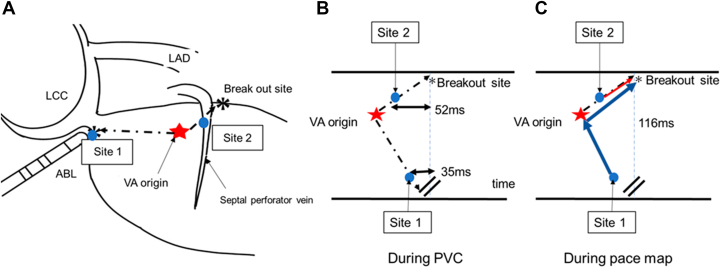
Conceptual Illustration of Preferential Conduction in an Intramural Ventricular Arrhythmia (A) Schematic diagram showing the spatial relationship between the intramural ventricular arrhythmia (VA) origin and two endocardial recording sites: the junction of the left and right coronary cusps (Site 1) and the septal perforator vein (Site 2). (B) Conceptual activation sequence during spontaneous PVC. Site 2, located closer to the VA origin, records earlier activation (52 ms before QRS onset). In contrast, Site 1, positioned farther from the VA origin, demonstrates less prematurity relative to the QRS (35 ms before QRS onset). (C) During pace mapping, the stimulus-to-QRS latency is longest at Site 1, the most remote endocardial site from the breakout point, reflecting conduction through a preferential intramural pathway. ABL = ablation catheter; LAD = left anterior descending artery; LCC = left coronary cusp; PVC = premature ventricular contraction; VA = ventricular arrhythmia.

Notably, subtle discrepancies were observed in the right precordial leads (V_1_ and V_2_) compared with the clinical PVC. In contrast, pacing from the septal perforator vein resulted in an almost identical match across all leads, supporting direct capture of myocardium adjacent to the true intramural arrhythmogenic focus. During sinus rhythm, delayed discrete potentials at the LCC/RCC junction further supported the presence of a concealed preferential conduction pathway. The proposed mechanism is schematically illustrated in Figure 5.

Coronary venous mapping using a microelectrode catheter proved critical in localizing the true arrhythmogenic substrate.^5^, ^6^, ^7^ The earliest activation was recorded within a septal perforator vein, consistent with an intramural origin and preferential conduction to a distant myocardial breakout site. Although intramural substrates are well recognized in idiopathic outflow tract arrhythmias, their contribution to ablation failure may be underappreciated when preferential conduction is present. Importantly, this electrophysiological behavior should not be generalized to all intramural ventricular arrhythmias; however, when a good endocardial pace map is accompanied by marked pacing latency, a remote intramural focus should be suspected.

Because effective lesion delivery from the endocardial surface was unlikely, chemical ablation was selected. In the present case, endocardial ablation was performed without an intraseptal mapping catheter in place; therefore, any potential augmentation of intramural lesion formation due to metallic heating could not be used. Prior experimental studies have demonstrated that radiofrequency energy delivery in proximity to metallic components may enhance intramural lesion formation, which could potentially influence ablation efficacy in selected intramural septal substrates.^8^ This technical consideration may partly explain the failure of endocardial ablation in the present case.

Retrograde coronary venous ethanol ablation has emerged as a useful strategy for intramural or inaccessible substrates^9^; however, safety concerns, particularly atrioventricular block, remain when targeting septal veins. In this context, the double-balloon technique^10^ offered a distinct advantage by allowing selective ethanol delivery confined to the target segment while preventing distal spread and collateral washout. This approach enabled effective ablation with a minimal ethanol dose and without conduction system injury.

Importantly, although this arrhythmia exhibited features overlapping with left ventricular summit VA, the primary mechanism was intramural rather than epicardial. This distinction underscores the need to move beyond anatomical labels and instead integrate electrophysiological characteristics, particularly pacing latency and prepotential behavior, to guide management.

**Visual Summary:**
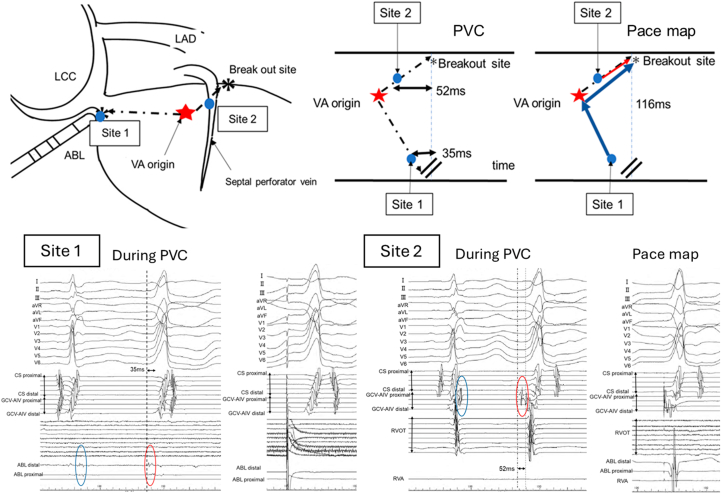
Intramural Ventricular Arrhythmia With Preferential Conduction: Diagnostic and Therapeutic Approach Electrocardiographic findings suggested an epicardial outflow tract ventricular arrhythmia. Endocardial mapping revealed a preferential conduction pathway with a remote breakout site, resulting in misleading pace-mapping findings. Coronary venous mapping identified the true intramural arrhythmogenic substrate. Selective ethanol ablation using a double-balloon technique achieved durable elimination of the ventricular arrhythmia. ABL = ablation catheter; AIV = anterior interventricular vein; CS = coronary sinus; GCV = great cardiac vein; LAD = left anterior descending artery; LCC = left coronary cusp; PVC = premature ventricular contraction; RVA = right ventricular apical; RVOT = right ventricular outflow tract; VA = ventricular arrhythmia.

## Conclusions

This case demonstrates that intramural ventricular arrhythmias with preferential conduction can masquerade as epicardial or left ventricular summit arrhythmias, leading to failure of conventional ablation strategies. Careful assessment of pacing latency, prepotential behavior, and coronary venous mapping can enable accurate identification of intramural substrates. Selective ethanol ablation using a double-balloon technique provides an effective and safe therapeutic option when endocardial ablation is unsuccessful.

## Funding Support and Author Disclosures

The authors have reported that they have no relationships relevant to the contents of this paper to disclose.
